# A hybrid interpretable deep structure based on adaptive neuro-fuzzy inference system, decision tree, and K-means for intrusion detection

**DOI:** 10.1038/s41598-022-23765-x

**Published:** 2022-12-01

**Authors:** Jia Liu, Wang Yinchai, Teh Chee Siong, Xinjin Li, Liping Zhao, Fengrui Wei

**Affiliations:** 1 Faculty of Computer Science and Technology, University Malaysia Sarawak, 89007 Sarawak, Malaysia; 2 School of Big Data, Weifang Institute of Technology, Weifang, 262500 China; 3 Faculty of Cognitive Sciences and Human Development, University Malaysia Sarawak, 89007 Sarawak, Malaysia; 4 College of Control Science and Engineering, China University of Petroleum, Qingdao, 266580 China

**Keywords:** Applied mathematics, Computational science, Computer science, Information technology

## Abstract

For generating an interpretable deep architecture for identifying deep intrusion patterns, this study proposes an approach that combines ANFIS (Adaptive Network-based Fuzzy Inference System) and DT (Decision Tree) for interpreting the deep pattern of intrusion detection. Meanwhile, for improving the efficiency of training and predicting, Pearson Correlation analysis, standard deviation, and a new adaptive K-means are used to select attributes and make fuzzy interval decisions. The proposed algorithm was trained, validated, and tested on the NSL-KDD (National security lab–knowledge discovery and data mining) dataset. Using 22 attributes that highly related to the target, the performance of the proposed method achieves a 99.86% detection rate and 0.14% false alarm rate on the KDDTrain+ dataset, a 77.46% detection rate on the KDDTest+ dataset, which is better than many classifiers. Besides, the interpretable model can help us demonstrate the complex and overlapped pattern of intrusions and analyze the pattern of various intrusions.

## Introduction

The leading technologies increase the cyber risk for users and businesses. According to *Cisco Annual Internet Report (2018–2023) White Paper*^1^, the threat of network intrusions continues to grow. This report illustrated that there was a 776% growth in attacks between 100 and 400 Gbps from 2018 to 2019. And, over half of the operators experienced infrastructure outages. The advance in technologies such as e-commerce, mobile payments, cloud computing, Big Data and analytics, IoT, AI, machine learning, and social media is the main driver of economic growth but has also led to a higher incidence of cyberattacks. As one of the key technologies for ensuring network security, intrusion detection plays a more important role. In the new network environment, new technologies need to be studied to improve the intrusion detection methods.

**Figure 1:**
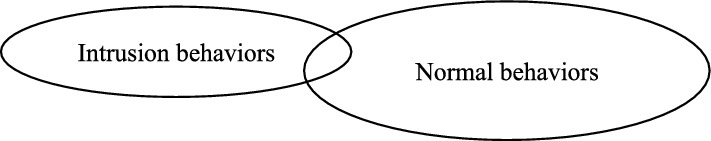
The overlap between intrusion behaviours and normal behaviours.

Because the behaviors of intruders are bound to overlap with that of legitimate users, the overlap between normal behaviors and abnormal behaviors is uncertain, as shown in Fig. 1. Therefore, intrusion detection problems are fuzzy classification problems to some extent. To get better performance on intrusion detection, we need to adapt to the fuzzy characteristic of intrusion detection. Fuzzy logic is a choice. Fuzzy schemes have successfully detected intrusions and malicious behaviors in the presence of uncertain data^2^. A large number of fuzzy approaches have been successfully applied in IDSs (intrusion detection systems). However, in many fuzzy approaches used in intrusion detection, Mohammad Masdari and Hemn Khezri^2^ illustrated that the ANFIS classifier is one mostly used classifier.

ANFIS is an algorithm that combines the uncertainty processing ability of fuzzy logic with the learning process of the ANNs (Artificial Neural Networks). ANFIS was first used in IDSs in 2007, in which Toosi and Kahani^3^ used five ANFIS modules to explore intrusive activity, which reaching a 95.3% detection rate on the KDDCUP99 dataset. However, ANFIS algorithm has its own disadvantage. It only has five layers, resulting in the disability of identifying deep features. Meanwhile the advantage of ANFIS is also outstanding. It can generate fuzzy and overlapped fuzzy rules and use the parameters learning method like ANNs. Since it only has five layers, which make the architecture of ANFIS is more interpretable than deep ANNs.

So, for generating a more interpretable and deep structure for IDS, we combine the ANFIS and CART (classification and regression tree) to co-train and identify the deep intrusion patterns. To improve the training and predicting efficiency of the proposed algorithm, we use Pearson Correlation analysis, standard deviation, and a new adaptive K-means are used to select attributes and make fuzzy interval decisions, to minimize the number of fuzzy rules.

## Related work

Norbert Wiener, the founder of cybernetics, pointed out that man's superiority over the most perfect machine is that man is capable of using fuzzy concepts. This shows that there is an essential difference between the human brain and the computer. This also shows how important fuzzy logic can be in simulating the human brain and dealing with fuzzy problems.

**Figure 2:**
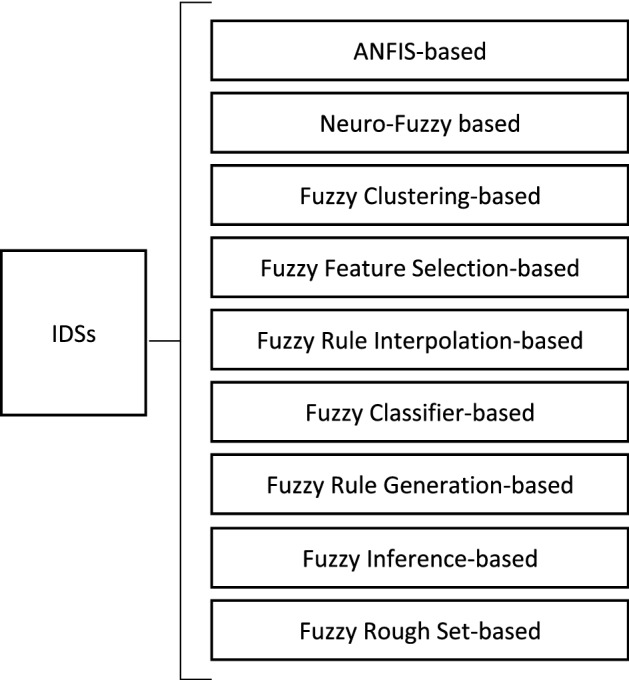
Classification of fuzzy IDSs.

Fuzzy schemes have successfully detected intrusions and malicious behaviours in the presence of uncertain data^2^. Therefore, a large number of fuzzy approaches have been successfully applied in IDSs. Mohammad Masdari and Hemn Khezri^2^ categorized various fuzzy intrusion detection schemes into nine categories, as shown in Fig. 2. They also illustrated that the ANFIS classifier is one mostly used classifier in various misuse detection schemes.

ANFIS algorithm is an algorithm that combines the uncertainty processing ability of fuzzy logic with the learning process of the ANNs. ANFIS was first used in IDSs in 2007. Toosi and Kahani^3^ used five ANFIS modules to explore intrusive activity, which reaching a 95.3% detection rate on the KDDCUP99 dataset.

Then, Chan et al.^4^ presented a policy-enhanced fuzzy model with ANFIS characteristics. Devi et al.^5^ introduced an IDS scheme using ANFIS to detect security attacks on 5G wireless networks.

In combination with other algorithms, Karaboga and Kaya^6^ proposed a hybrid artificial bee colony (ABC) algorithm to train ANFIS. This algorithm uses arithmetic crossover to converge quickly r and has better efficiency than the standard ABC algorithm.

Altyeb Altaher presented an IDS scheme EHNFC in^7^, which is an evolutionary neuro-fuzzy classifier for malware classification. It can use fuzzy rules to detect fuzzy malware and improve its detection accuracy by learning new fuzzy rules to evolve its structure. In addition, it uses an improved fuzzy rule updating clustering method to update the centroid and radius of the clustering permission feature. These changes to the application of clustering methods improve the convergence of clustering and create rules that adapt to the input data, thereby improving the accuracy.

The scheme introduced in [8] adopts data mining methods such as neural fuzzy and radial basis support vector machine to achieve a high detection rate when dealing with security attacks. The method is mainly divided into four stages, and k-means clustering is used to generate parameter-tuning subsets. Based on these subsets, various neural fuzzy models are trained to form classification vectors to support vector machines.

For solving the problem of the large volume of data resulting in the network getting expanded with false alarm rate of intrusion while decreased in detection accuracy, Manimurugan et al.^9^ presented an algorithm CSO-ANFIS. This algorithm uses the Crow Search Optimization algorithm to optimize ANFIS and reaches a 95.8% detection rate on the NSL-KDD (National security lab–knowledge discovery and data mining) dataset.

In summary, ANFIS is widely used and studied in intrusion detection systems and misuse detection systems. And, studies that combine with other algorithms had been conducted extensively to get a higher detection rate. However, one disadvantage of ANFIS is the limited layers, which results in the hardness of identifying deeper patterns of fuzzy problems, such as intrusion detection problems.

## Preliminaries: ANFIS and CART

### Adaptive neuro-fuzzy inference system*—ANFIS*

**Figure 3:**
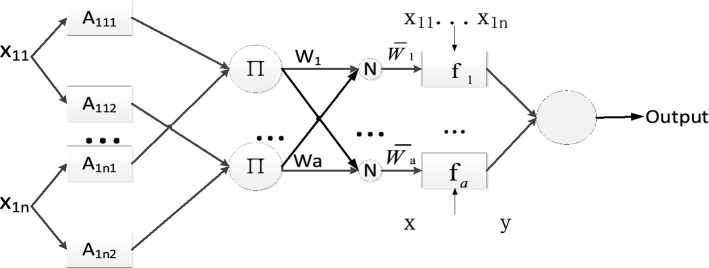
Architecture of ANFIS.

ANFIS algorithm combines the uncertainty processing ability of fuzzy logic with the learning process of the ANNs. The basic structure of most fuzzy inference systems (FISs) is a model that maps the input characteristics to the input membership functions (MF). Rather than choosing arbitrarily the parameters of membership functions, ANFIS uses a hybrid learning methodology like ANNs to learn these parameters so that these parameters can customize the membership function based on variations in input/output data. It is especially important for systems where the properties are not fully understood by humans or where the properties are complex. It is a multilayer feed forward network consisting of five layers. Each layer in the ANFIS is shown in Fig. 3.

The five layers of Gaussian ANIFIS are:

**Layer 1**: Calculate the membership grade of the inputs with the Gaussian membership function (MF).
  1 $$\mu_{{A_{1nj} }} (x_{1n} ) = e^{{ - (\frac{{x{\text{-}}mean_{n} }}{{sigma_{n} }})^{2} }}$$
  Where ${x}_{1n}$ is the input to the node ${A}_{1n}$. $n$ is the number of attributes. $j$ is the linguistic label (small, large, etc.) of attribute $n$. ${A}_{1nj}$ is the membership that determines the degree to which input ${x}_{1n}$ satisfies ${A}_{1nj}$. ${mean}_{n}$ represents the center of the Gaussian function. ${sigma}_{n}$ represents the width of the Gaussian function. ${mean}_{n}$ and ${sigma}_{n}$ are the learning parameters. They are referred to as premise parameters. As the values of these parameters change, the Gaussian functions vary accordingly.
**Layer 2:** Firing strengths. Each node in this layer calculates the firing strength of the $i$-th rule.
  2 $$w_{i} = \mu_{{A_{11j} }} (x_{11} ) \times \cdots \times \mu_{{A_{1nj} }} (x_{1n} )$$
  Where $i$ is the node number of Layer2. Each node output represents the firing strength of a rule.
**Layer 3:** Calculate the normalized firing strength of the $i$-th rule.
  3 $$\overline{w}_{i} = \frac{{w_{i} }}{{\sum\nolimits_{i = 1}^{a} {w_{i} } }}$$
  Where a is the number of nodes in Layer2.
**Layer 4:** Adaptive node with a linear function. Each node calculates the weighted value of the consequent part of each rule.
  4 $$\overline{w}_{i} f_{a} = \overline{w}_{i} (p_{i} x + q_{i} y + r_{i} )$$
  Where ${p}_{i}, {q}_{i}, {r}_{i}$ are the learning parameters. These parameters are referred to as consequent parameters.
**Layer 5:** Produce the overall output by aggregating all the fired rule values.
  5 $$output = \sum\limits_{i} {\overline{w}_{i} \cdot f_{i} }$$

The main learning parameters of ANFIS are premise parameters and consequent parameters. ANFIS provides 4 methods to update the parameters:

- ***Gradient descent only***: All parameters are updated by gradient descent.
- ***Gradient descent and one pass of LSE***: The $LSE$ is applied only once at the beginning to get the initial values of the consequent parameters and then the gradient descent takes over to update all parameters.
- ***Gradient descent and LSE***^10^: This is the proposed parameters updating method in the original ANFIS. In the forward-passing process of hybrid learning, the node output is propagated to the fourth layer, and the least-squares method is used to estimate the consequent parameters. In backward propagation, the error (the difference between the expected output and the actual output) is propagated back to the first layer, with the premise parameters updated using gradient descent, while the consequent parameters are fixed.
- ***Sequential (approximate) LSE only***: The premise parameters and the extended Kalman filter algorithm are employed to update all parameters.

### Classification and regression tree*—CART*

CART is a two-branches decision tree that uses the Gini index. The Gini index is an inequality measure between 0 and 1 that can be used to measure any uneven distribution to select the optimal feature. The Gini index is 0 means equal and 1 means completely different. When we measure the Gini index of all values belonging to a feature of the data set, we can obtain the Gini index of that feature. The process of recursively creating a classification decision tree is to select the GiniGain of the smallest node as the branch point until the subset belongs to the same class or all features are used. For a $K$ class, the calculation of the CART algorithm is as follows.

6 $$Gini(p) = \sum\limits_{k = 1}^{K} {p_{k} (1 - p_{k} )} = 1 - \sum\limits_{k = 1}^{K} {p_{k}^{2} }$$

Where ${p}_{k}$ is the probability that the sample point belongs to the $k$-th class_._ $Gini(p)$ is the Gini index of the probability distribution.

7 $$Gini(D) = 1 - \sum\limits_{k = 1}^{K} {\left( {\frac{{\left| {C_{k} } \right|}}{\left| D \right|}} \right)}^{2}$$

Where $Gini(D)$ is the Gini index of sample set $D$. ${C}_{k}$ represents the sample subset belonging to the $k$-th class in data set $D$.

If data set $D$ is segmented on feature $A$’s certain value $a$, obtaining parts ${D}_{1}$ and ${D}_{2}$. The Gini index of set $D$ under feature $A$ is shown below.

8 $$Gain\_Gini(D,A) = \frac{{\left| {D_{1} } \right|}}{\left| D \right|}Gini(D_{1} ) + \frac{{\left| {D_{2} } \right|}}{\left| D \right|}Gini(D_{2} )$$

Where $Gain\_Gini(D, A)$ represents the uncertainty of set $D$ after $A=a$ segmentation. The greater the value, the greater the uncertainty of the sample set.

For feature $A$, the $Gain\_Gini$ after dividing the dataset into two parts by every feature value is calculated respectively, and the minimum value is selected as the optimal binary scheme obtained by feature $A$.

9 $$\mathop {\min }\limits_{i \in A} (Gain\_Gini(D,A))$$

Then, for sample set $D$, the optimal binary scheme of all features is calculated, and the minimum value is selected as the optimal binary scheme of sample set $D$.

10 $$\mathop {\min }\limits_{A \in Attribute} (\mathop {\min }\limits_{i \in A} (Gain\_Gini(D,A)))$$

## Proposed methodology

In the proposed method, ANFIS is used to generate the overlapped and fuzzy rules (fuzzy and overlapped combinations of attributes’ fuzzy intervals). CART is used to deeply extract and interpret the patterns in the data. Meanwhile, for improving the training and predicting efficiency, correlation analysis, standard deviation, and a proposed adaptive K-means algorithm are used to select attributes and decide attributes’ fuzzy intervals. So, one of the key differences between our work and other papers is the interpretable, deep structure for intrusion detection. The other is the efficiency improvement.

### Attributes selection

The purpose of attribute selection is to reduce the dimension of data. Due to ANFIS uses multiplying all the incoming signals to form rules in Layer2, it results in a large number of generated rules. Therefore, it is necessary to conduct preliminary attribute selection for problems with many attributes.

We used Pearson correlation analysis to select attributes in the proposed method. The Pearson correlation coefficient $r$ is usually used to measure whether there is a linear relationship between attributes as below:

11 $${\text{r}}_{xy} = \frac{{\sum {(x - \overline{x})} (y - \overline{y})}}{{\sqrt {\Sigma_{1}^{n} (x_{i} - \overline{x})^{2} } \sqrt {\Sigma_{1}^{n} (y_{i} - \overline{y})^{2} } }}$$

The $r$ describes the degree of linear correlation between $x$ and $y$. $r>0$ indicates $x$ and $y$ are positively correlated. $r<0$ indicates $x$ and $y$ are negatively correlated. The larger the absolute value of $r$, the stronger the correlation. In normal circumstances, the correlation strength of the attribute is judged by the following value range: 0.8–1.0 very strong correlation, 0.6–0.8 strong correlation, 0.4–0.6 moderate correlation, 0.2–0.4 weak correlation, 0.0–0.2 very weak correlation or no correlation. In this study, we use 0.1 as the minimum threshold for selecting attributes.

The continuous attributes selected by Pearson correlation analysis are used as the inputs to the ANFIS. An important advantage of using correlation analysis to select continuous attributes is that the selected continuous attributes have a higher degree of correlation with the target and a lower data dimension.

### Standard deviation analysis and adaptive K-means

Standard deviation is used to determine the original interval number of continuous attributes selected by Pearson correlation analysis. Adaptive K-means is used to finally minimize and check the interval number of continuous attributes.

Standard deviation reflects the degree of dispersion of an attribute. The larger the standard deviation is, the more intervals are needed to represent the dispersion. Therefore, in this study, we related the original number of attribute intervals to the exponent of the standard deviation, which used the exponent of the standard deviation of each selected continuous attribute plus 2 as the original interval number.

Meanwhile, to further minimize and check the number of intervals, we proposed an adaptive K-means to dynamically determine the number of intervals.

K-means is one of the most popular clustering algorithms because it is very flexible, simple, intuitive, easy to implement, and fast in execution^11^. Although K-means is a useful clustering algorithm, it also has some disadvantages. One disadvantage is the number of clusters $k$ of K-means must be known in advance. The main steps of the K-means clustering algorithm are as follows:

**Stage 1**: According to the data ranges of $n$ data objects, $k$ cluster centers are randomly initialized.
**Stage 2**: Assign each object to the group closest to the center.
**Stage 3**: The location of each center is updated by calculating the average of the objects assigned to it.
**Stage 4**: Stage 2 and Stage 3 are repeated until the maximum number of iterations is reached, or until the cluster center is no longer moving.

**Figure 4:**
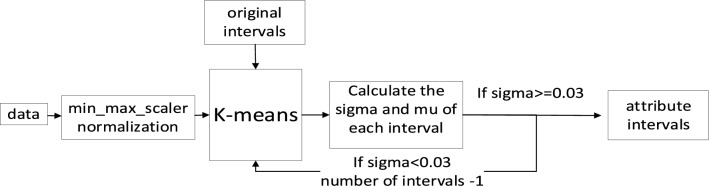
Adaptive K-means algorithm.

To dynamically determine the minimum number of clusters $k$. An adaptive K-means algorithm is proposed (see Fig. 4).

This method is based on the following four stages:

**Stage 1**: Min_max_scaler normalization is used to normalize all attributes. This is to eliminate the dimensional influence among attributes.
**Stage 2**:  K-means is used to calculate the cluster centers of each interval. The original interval number of K-means is calculated from the standard deviation, which is the exponent of the standard deviation of each attribute plus 2.
**Stage 3**: Divide the data into intervals and calculate the $sigma$ and $mean$ of each interval as below. If one interval’s $sigma$ is less than 0.03 which means insufficient dispersion of data on this interval, the clustering number will be reduced by 1, and the clustering is returned to Stage2 for re-clustering.
  12 $$mean = \frac{{\sum\nolimits_{1}^{n} {x_{i} } }}{n}$$
  13 $$sigma = \sqrt {\frac{{\sum\nolimits_{1}^{n} {(x_{i} - mean)^{2} } }}{n}}$$
  Where $n$ is the total amount of data.
**Stage 4**: If the $sigma$ of each attribute is greater than 0.03, the attribute is finally distributed according to the clustering intervals. And, the calculated $sigma$ and $mean$ of intervals will be the original $sigma$ and $mean$ before being trained by the proposed method, which will make the $sigma$ and $mean$ of the ANFIS at an appropriate level during the initial phase.

### Connection of ANFIS and CART

**Figure 5:**
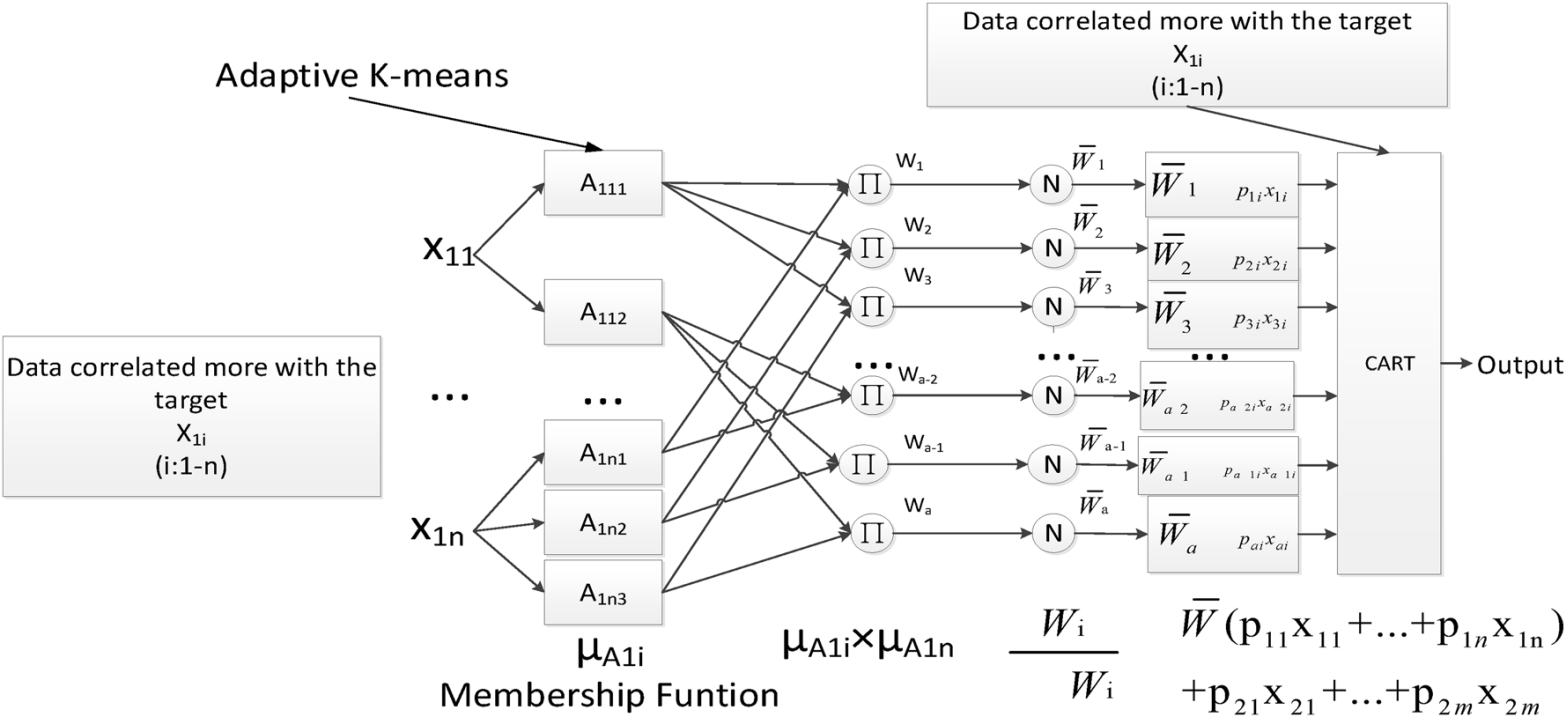
Modified ANFIS for generating weighted rules.

For making full use of the advantage of ANFIS and CART, to form interpretable, self-adaptive parameters, and deep structure for intrusion detection, we concatenate the ANFIS and CART as shown in Fig. 5.

In Fig. 5, the intervals of the selected attribute ${x}_{1i}$ are divided according to the standard deviation and adaptive K-means, which will generate overlapped intervals. Then, the membership function is used to turn the value of these attributes into the membership degree belonging to the intervals. By not just providing the exact continuous value, but the membership degree of fuzzy intervals (e.g., big, medium, small), these membership degrees will provide certain fuzzy, overlapped, and generalization abilities for the proposed algorithm. Then, fuzzy rules (product of different interval membership degrees of attributes) are generated to represent different possible combinations of attributes (such as duration is small, count is medium, diff_srv_rate is big). After the normalization of all the ${w}_{i}$ (rules’ firing strengths), weighted fuzzy rules are formed, which represent the rules’ contribution to the target. Finally, the weighted fuzzy rules and original ${x}_{1i}$ will be concatenated to input CART for interpreting the deep patterns.

The layers in the proposed method are shown below:

**Layer 1:** According to the $means$ and $sigmas$ calculated by adaptive K-means to form the Gaussian membership function (MF). And, calculate the membership grade of the inputs with the MFs.
  14 $$\mu_{{A_{1nj} }} (x_{1n} ) = e^{{ - (\frac{{x{\text{-}}mean_{n} }}{{sigma_{n} }})^{2} }}$$
  Where $mean$ and $sigma$ are the learning parameters.
**Layer 2:** Firing strengths. Each node in this layer calculates the firing strength of the $i$-th rule.
  15 $$w_{i} = \mu_{{A_{11j} }} (x_{11} ) \times \cdots \times \mu_{{A_{1nj} }} (x_{1n} )$$
**Layer 3:** Calculate the normalized firing strength of the $i$-th rule.
  16 $$\overline{w}_{i} = \frac{{w_{i} }}{{\sum\nolimits_{i = 1}^{a} {w_{i} } }}$$
  Where a is the number of rules.
**Layer 4:** $WF=\{{WF}_{1},{WF}_{2},\ldots ,{WF}_{a}\}, {WF}_{i}=\left\{\overline{{w }_{i}}{f}_{1},\overline{{w }_{i}}{f}_{2},\ldots ,\overline{{w }_{i}}{f}_{d}\right\}$. Weighted rules set.
  17 $$\overline{w}_{i} f_{sel\_i} = \overline{w}_{i} p_{sel\_i} x_{sel\_i}$$
  Where ${p}_{sel\_i}$ is the learning parameter.
**Layer 5:** Concatenate the weighted rules, original ${X}_{1n}$ as inputs of CART.
  18 $${X}^{^{\prime}}=Concatenate(\mathrm{WF},{X}_{1n})$$
**Layer 6:** Calculate the result ${Y}^{^{\prime}}$ by CART. Then, calculate and feedback the gradient to train the learning parameters in ANFIS. Because the computation complexity of the least squares estimate is higher than that of the gradient descent. Therefore, we update the learning parameters (premise parameters and consequent parameters) all by gradient descent. The actual algorithm is shown in Algorithm 1.

**Algorithm 1** The proposed algorithm

- **Input**: ${X}_{sel}\in {\mathbb{R}}^{m\times d}$, ${X}_{sel}$ is selected attributes.
- **Output**: Classification result $Y\in {\mathbb{R}}^{m}$.
- Step 1. $${\varvec{A}}{\varvec{N}}{\varvec{F}}{\varvec{I}}{\varvec{S}}({X}_{sel})\to (WF)$$:
  - The original Gaussian membership functions (formed by $means$ and $sigmas$ after Adaptive K-means).
    19 $$\mu (x) = e^{{ - \frac{1}{2}(\frac{x{\text{-}}mean}{{sigma}})^{2} }}$$
    Where $means$ and $sigmas$ are the learning parameters.
  - ${w}_{i}$, firing strengths of $i$-th rule, as follows:
    20 $$w_{i} = \mu_{{A_{11j} }} (x_{11} ) \times \cdots \times \mu_{{A_{1nj} }} (x_{1n} )$$
  - $\overline{{w }_{i}}$, firing strengths of $i$-th rule, as follows:
    21 $$\overline{w}_{i} = \frac{{w_{i} }}{{\sum\nolimits_{i = 1}^{a} {w_{i} } }}$$
    Where $a$ is the number of rules.
  - $WF=\{{WF}_{1},{WF}_{2},\cdots ,{WF}_{a}\}, {WF}_{i}=\left\{\overline{{w }_{i}}{f}_{1},\overline{{w }_{i}}{f}_{2},\cdots ,\overline{{w }_{i}}{f}_{d}\right\}$, weighted rules set, each weighted rule can be calculated as follows:
    22 $$\overline{w}_{i} f_{sel\_i} = \overline{w}_{i} P_{sel\_i} X_{sel\_i}$$
    Where ${X}_{sel\_i}\epsilon {X}_{sel}, sel\_i=\mathrm{1,2},\ldots ,m$. ${P}_{sel\_i}=\left\{{p}_{1},{p}_{2},\ldots {p}_{d}\right\}$, ${P}_{sel\_i}$ are the learning parameters.
- Step 2. $${\varvec{C}}{\varvec{A}}{\varvec{R}}{\varvec{T}}(WF,{X}_{sel})\to({Y}^{{\prime}})$$:
  - Concatenate $$WF,{X}_{sel}$$ as:
    23 $${X}^{^{\prime}}=(WF,{X}_{sel})$$
  - Calculate ${\varvec{C}}{\varvec{A}}{\varvec{R}}{\varvec{T}}({X}^{^{\prime}})$ to get ${Y}^{^{\prime}}$.
- Step 3. $${\varvec{B}}{\varvec{a}}{\varvec{c}}{\varvec{k}}{\varvec{p}}{\varvec{r}}{\varvec{o}}{\varvec{p}}{\varvec{a}}{\varvec{g}}{\varvec{a}}{\varvec{t}}{\varvec{i}}{\varvec{o}}{\varvec{n}}({Y}^{^{\prime}},Y)$$:
  - Calculate the MSE loss of $({Y}^{^{\prime}},Y)$. Then, calculate the gradient with the function in Layer4. Use SGD (learning rate = 1e−4, momentum = 0.99) to update the premise parameters and consequent parameters.
- Step 4. $${\varvec{T}}{\varvec{r}}{\varvec{a}}{\varvec{i}}{\varvec{n}}{\varvec{i}}{\varvec{n}}{\varvec{g}}\;{\varvec{a}}{\varvec{n}}{\varvec{d}} \; {\varvec{V}}{\varvec{e}}{\varvec{r}}{\varvec{i}}{\varvec{f}}{\varvec{y}}\_{\varvec{L}}{\varvec{o}}{\varvec{s}}{\varvec{s}}({Y}^{^{\prime}},Y)$$:
  - Keep training the algorithm from Step 1 to Step 3.
  - Calculate the loss of $({Y}^{{\prime}},Y)$ on each epoch.
  - Early Stopping mechanism (patience = 5). If the loss keeps no improvement after 5 epochs, stop training.

## Results

We used the NSL-KDD dataset as the experimental dataset to test the proposed method with six metrices (precision, recall rate, F1-Score, accuracy, detection rate, and false alarm rate). And, after the attribute selection, 22 of the 41 attributes are selected as the experimental attributes, which reduces the dimension of data. Then, though standard deviation analysis and adaptive K-means, the number, $means$ and $sigmas$ of each attribute’s intervals are initialized. Then, the algorithm is trained as shown in Algorithm 1, reaching 99.86% DR (detection rate) and a 0.14% FAR (false alarm rate) on KDDTrain+ dataset.

### Data description

The simulation and performance analysis was carried out using Python and sklearn. The simulation used the NSL-KDD dataset^12^ as the training and testing dataset.

NSL-KDD (National security lab–knowledge discovery and data mining) is the enhanced form of KDD99 to outperform its limitations. It is a traditional benchmark dataset in intrusion detection. The most commonly used subset of NSL-KDD is the KDDTrain+ subset and the KDDTest+ subset. The traffic distribution of the KDDTrain+ subset and KDDTest+ subset is different. Some new intrusion types exist only in the KDDTest+ subset. So, KDDTest+ can be used to test the generalization ability of algorithms.

The KDDTrain+ subset contains 23 classes, including 22 types of attacks and normal. The KDDTest+ contains 38 classes, including 37 types of attacks and normal. The traffic distribution of KDDTrain+ and KDDTest+ is shown in Table 1.

**Table 1:** Distribution of five major categories of behaviours.

Category	KDDTrain+ dataset		KDDTest+	
	Count	Percentage (%)	Count	Percentage (%)
Normal	67,342	53.46	9711	43.08
Probe	45,927	36.46	7457	33.08
DOS	11,656	9.25	2754	12.22
U2R	995	0.79	2421	10.74
R2L	52	0.04	200	0.89
Total	125,972	100	22,543	100

The NSL-KDD dataset has 43 attributes, including 41 attributes, 1 target, and 1 hard attribute (hard attribute is used to describe the hard degree of classification). Of the 41 attributes, 7 are discrete and 34 are continuous.

In this study, an 80% KDDTrain+ subset was used as the training dataset. The full KDDTrain+ subset and KDDTest+ subset are served as the validation and test set, respectively.

### Performance metrics

The proposed method will be evaluated by precision, recall rate, F1-Score, ACC (accuracy), DR (detection rate), and FAR (false alarm rate). These metrics can be calculated by TP, TN, FP, and FN, as shown in Table 2.

**Table 2:** Four situations of results.

Sample	Classification	
	Positive	Negative
Positive	True positive (TP)	False negative (FN)
Negative	False positive (FP)	True negative (TN)

Because samples containing attacks can be defined as positive samples, while samples without attacks can be defined as negative samples in intrusion detection. All possible results can be divided into the following four situations: TP, TN, FP, and FN.

*Precision:* The percentage of all TP samples to all positive classifications (TP + FP).

24 $$\Pr ecision = \frac{TP}{{TP + FP}}$$

*Recall rate:* The percentage of all TP samples to all samples (TP + FN) that should be positive.

25 $${\text{Re}} call = \frac{TP}{{TP + FN}}$$

*Accuracy (ACC):* The percentage of all correct predictions.

26 $$ACC = \frac{TP + TN}{{TP + FN + FP + FN}}$$

*F1-score:* Harmonic mean of precision and recall rate. When the precision and recall rate are high, the F1-Score is high. A high F1-Score indicates high precision and recall. When the precision and recall are both 100%, the best F1-Score is 1. The worst F1-Score is 0. The F1-Score is a measure of test accuracy.

27 $$F_{1} = 2 \times \frac{{\Pr ecision \times {\text{Re}} call}}{{\Pr ecision + {\text{Re}} call}}$$

*Detection rate:* The percentage of successfully categorizing this data in this category.

28 $$DR = \frac{TP}{{TP + FN}}$$

*False alarm rate (FAR):* The percentage of data that should not be identified for this category.

29 $$FAR = \frac{FP}{{TN + FP}}$$

### Experimental results

Through Pearson correlation analysis, 22 (absolute value of correlation degree is greater than 0.1, more related to target) of the 41 attributes are selected as the experimental attributes. They are duration, protocol_type, service, flag, logged_in, count, serror_rate, srv_serror_rate, rerror_rate, srv_rerror_rate, same_srv_rate, diff_srv_rate, dst_host_count, dst_host_srv_count, dst_host_same_srv_rate, dst_host_diff_srv_rate, dst_host_same_src_port_rate, dst_host_srv_diff_host_rate, dst_host_serror_rate, dst_host_srv_serror_rate, dst_host_rerror_rate and dst_host_srv_rerror_rate. The correlation degree formed by Pearson correlation analysis is shown in Table 3. The entire correlation matrix of 42 attributes can be found as Supplementary Fig. S1 online.

**Table 3:** Correlation degree of 41 attributes towards target.

Attribute	Correlation degree
dst_host_srv_count	− 0.62425
logged_in	− 0.57604
hard	− 0.557252
dst_host_same_srv_rate	− 0.525837
same_srv_rate	− 0.510869
flag	− 0.501333
srv_count	− 0.038856
num_access_files	− 0.030234
su_attempted	− 0.019483
num_file_creations	− 0.014385
num_root	− 0.01002
num_compromised	− 0.009003
is_host_login	− 0.002334
land	0.002944
urgent	0.003941
num_shells	0.005208
root_shell	0.007757
dst_bytes	0.008651
srv_diff_host_rate	0.010454
src_bytes	0.012981
num_failed_logins	0.02312
is_guest_login	0.0306
wrong_fragment	0.054795
hot	0.065085
duration	0.129443
protocol_type	0.168167
dst_host_count	0.196101
service	0.233783
dst_host_srv_diff_host_rate	0.239645
diff_srv_rate	0.282313
dst_host_rerror_rate	0.293804
rerror_rate	0.308667
srv_rerror_rate	0.309907
dst_host_srv_rerror_rate	0.310226
dst_host_same_src_port_rate	0.319097
srv_serror_rate	0.378666
serror_rate	0.381644
dst_host_serror_rate	0.382115
dst_host_srv_serror_rate	0.384793
count	0.388355
dst_host_diff_srv_rate	0.391327

Through standard deviation analysis, the original interval number of attributes is shown in Table 4.

**Table 4:** The standard deviation and original interval number of attributes.

Attribute name	Standard deviation	Original interval number
dst_host_srv_diff_host_rate	1.13E−01	1
diff_srv_rate	1.80E−01	1
dst_host_diff_srv_rate	1.89E−01	1
dst_host_rerror_rate	3.07E−01	1
dst_host_same_src_port_rate	3.09E−01	1
dst_host_srv_rerror_rate	3.19E−01	1
rerror_rate	3.20E−01	1
srv_rerror_rate	3.24E−01	1
same_srv_rate	4.40E−01	1
dst_host_serror_rate	4.45E−01	1
dst_host_srv_serror_rate	4.46E−01	1
serror_rate	4.46E−01	1
srv_serror_rate	4.47E−01	1
dst_host_same_srv_rate	4.49E−01	1
logged_in	4.89E−01	1
protocol_type	5.65E−01	1
flag	2.69E+00	1
service	1.69E+01	1
dst_host_count	9.92E+01	3
dst_host_srv_count	1.11E+02	4
count	1.15E+02	4
duration	2.60E+03	5

After the dynamical calculation of $sigmas$ and $means$ by the Adaptive K-means algorithm, the interval number of duration goes down to 4, and the final interval number of selected attributes is shown below. And the original $mean$ and $sigma$ for selected attributes (only list the original mean and sigma of attributes with interval number greater than 1) are shown in Table 5. Because the attributes with interval number equal to 1 do not involve overlap of intervals, their original sigma and mean are not listed.

**Table 5:** Final interval number of selected attributes.

Attribute name	Final interval number	Original mean and sigma (first is mean, second is sigma)
dst_host_srv_diff_host_rate	1	
diff_srv_rate	1	
dst_host_diff_srv_rate	1	
dst_host_rerror_rate	1	
dst_host_same_src_port_rate	1	
dst_host_srv_rerror_rate	1	
rerror_rate	1	
srv_rerror_rate	1	
same_srv_rate	1	
dst_host_serror_rate	1	
dst_host_srv_serror_rate	1	
serror_rate	1	
srv_serror_rate	1	
dst_host_same_srv_rate	1	
logged_in	1	
protocol_type	1	
flag	1	
service	1	
dst_host_count	3	[1.0, 0.9], [5.1, 1.3], [9.9, 0.4]
dst_host_srv_count	4	[0.4, 0.4], [2.9, 0.8], [6.2, 1.0], [9.9, 0.4]
count	4	[0.2, 0.2], [2.4, 0.6], [4.8, 0.7], [9.5, 0.8]
duration	4	[0.0, 0.1], [2.1, 0.7], [4.9, 0.9], [8.7, 0.9]

Only list the original mean and sigma of attributes with interval number greater than 1.

Through attribute selection (Pearson correlation analysis) and interval decision (standard deviation and adaptive K-means), the number of fuzzy rules generated by ANFIS is reduced to 192. This improves the training and predicting efficiency of the proposed algorithm.

The fuzzy rules obtained by ANFIS and original selected attributes are put into CART for extracting deep and interpretable patterns. The ANFIS and CART are co-train as shown in Algorithm 1. The training processes stop when the losses are not improved in 5 epochs. The final generated fuzzy rules (192 rules) with four attributes (number of attribute’s intervals greater than 1) of proposed method’s ANFIS-part can be found as Supplementary Table S1 online. The final coefficient parameter of 192 rules with four attributes (number of attribute’s intervals greater than 1) can be found as Supplementary Table S2 online.

The performance of the proposed method on the full KDDTrain+ subset and KDDTest+ subset is shown in Tables 6 and 7.

**Table 6:** Performance of proposed method on KDDTrain+ dataset.

Category	Precision (%)	Recall (%)	F1-score (%)	DR (%)	FAR (%)	ACC (%)
Normal	99.84	99.90	99.87	99.90	0.16	99.87
DOS	99.94	99.90	99.92	99.90	0.06	99.91
Probing	99.75	99.83	99.79	99.83	0.24	99.79
R2L	98.67	96.88	97.77	96.88	1.31	97.79
U2R	90.00	69.23	78.26	69.23	7.69	80.77
Weighted avg	99.86	99.86	99.86	99.86	0.14	99.86

**Table 7:** Performance of proposed method on KDDTest+ dataset.

Category	Precision (%)	Recall (%)	F1-score (%)	DR (%)	FAR (%)	ACC (%)
Normal	68.1	96.88	79.98	96.88	45.37	75.75
DOS	95.6	77.54	85.63	77.54	3.57	86.98
Probing	86.51	78.4	82.25	78.40	12.23	83.08
R2L	83.33	13.25	22.87	13.25	2.65	55.30
U2R	16.33	4.00	6.43	2.05	6.42	41.75
Weighted avg	80.58	77.46	74.46	77.46	22.54	77.46

### Interpret the intrusion patterns

Since the ANFIS only has five layers, the architecture of ANFIS is more interpretable than deep ANNs. The most important aspects of ANFIS are the generated rules and their contribution to the target. The equation of the weighted rules is shown below. In this equation, the firing strengths $\overline{{w }_{i}}$ are calculated by the combination of attributes’ fuzzy intervals, which represents the contribution of the rule to the target. A higher firing strength means a higher contribution to the target. The ${P}_{sel\_i}$ = $\left\{{p}_{1},{p}_{2},\ldots {p}_{d}\right\}$ represent the importance of the attributes to the rules. A higher ${p}_{i}$ means higher importance to the rule.

30 $$\overline{w}_{i} f_{sel\_i} = \overline{w}_{i} P_{sel\_i} X_{sel\_i}$$

Then, the CART part is more interpretable because the decision tree is an interpretable algorithm. We can easily read the importance of attributes or rules to the target.

**Figure 6:**
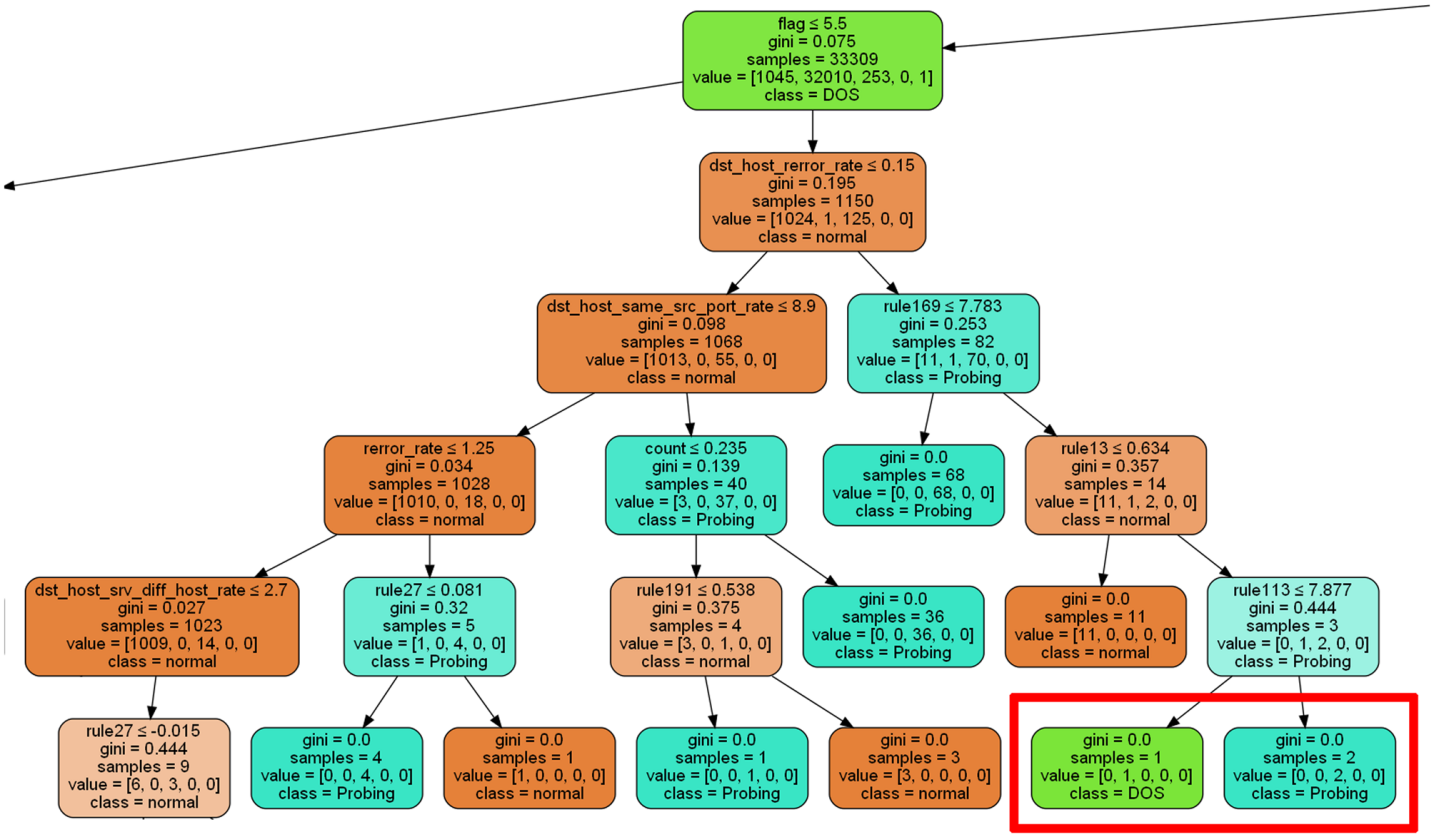
The node for interpreting the pattern.

We show one example to interpret the proposed method. If the results get DOS and Probing nodes in CART, as shown in Fig. 6. The entire generated CART tree can be found as Supplementary Fig. S2 online. From the generated complete CART tree, it can be seen that if same_srv_rate $\le$ 4.95, dst_host_diff_srv_rate $\le$ 1.55, flag $>$ 5.5, and dst_host_rerror_rate $>$ 0.15, rule169 > 7.783, rule13 $>$ 0.634, the behavior will be identified intrusion behavior. And, rule113 helps us identify intrusion types further. If rule113 $\le$ 7.877, the intrusion type is DOS, otherwise it is Probing. The rule167, rule13, and rule113 are all can be obtained from the complete model, as shown in Table 8. In this phase, the firing strength $\overline{{w }_{i}}$ will make different rule’s value zoom in or out of the distribution, which will influence the importance of the rule in the classification. The ${P}_{sel\_i}$ of rule will influence each attribute contribution to the rule.

**Table 8:** The classification pattern of the nodes.

Path	Dividing value
same_srv_rate	≤ 4.95
dst_host_diff_srv_rate	≤ 1.55
flag	> 5.5
dst_host_rerror_rate	> 0.15
rule169 (duration is 3, count is 2, dst_host_count is 0, dst_host_srv_count is 1)	> 7.783
rule13 (duration is 0, count is 1, dst_host_count is 0, dst_host_srv_count is 1)	> 0.634
rule113 (duration is 2, count is 1, dst_host_count is 1, dst_host_srv_count is 1)	> 7.877 (probing)
rule113 (duration is 2, count is 1, dst_host_count is 1, dst_host_srv_count is 1)	≤ 7.877 (DOS)

0–3 represent which fuzzy interval the attribute will be calculated in.

Therefore, using the combination and co-training mechanism of ANFIS and CART, we have a complete combination of attributes’ fuzzy intervals and get a deeper pattern extraction structure than just a single ANFIS or single CART. It helps us identify deeper patterns in intrusion detection. Particularly, the most important advantage of the proposed method is the pattern is more interpretable than ANNs, as we show above. It makes this algorithm more practical in engineering, which will help interpret the deep patterns of intrusions, defend against intrusions and establish engineering IDSs in the real world.

## Discussion

According to the experimental result on the KDDTrain+ dataset, the method presents a high detection rate and low false alarm rate. The DR (detection rate) on KDDTrain+ dataset reaches 99.86%. And the FAR (false alarm rate) on KDDTrain+ dataset reaches 0.14%.

On the KDDTest+ dataset, the proposed method also performs well, reflecting a certain generalization ability. The total DR reaches 77.46%, Precision reaches 80.58%, and ACC reaches 77.46%. However, the FAR on KDDTest+ is not ideal, accounting for 22.54%, which needs to be further improved. The DR in the Normal category reaches 96.88%. The Precision on DOS reaches 95.6%.

The performance of the proposed method was compared with other studies^9^. In terms of the classification performance of five main categories, the comparison of the performance with BPNN, GA-ANFIS, PSO-ANFIS, and CSO-ANFIS^9^ is shown in Tables 9 and 10. The comparison of the performance with single CART and ANFIS is shown in Table 11. The comparison of the accuracy with other existing approaches is shown in Table 12.

**Table 9:** Comparison of detection rate and FAR on KDDTrain+ dataset.

Classifier	DR	FAR
BPNN^9^	85.05	6.10
FC-ANN^9^	89.23	4.37
GA-ANFIS^9^	93.15	3.75
PSO-ANFIS^9^	94.46	3.38
CSO-ANFIS^9^	95.80	3.45
CART	99.74	**0.06**
Proposed Method	**99.86**	0.14

Significant values are in bold.

**Table 10:** Comparison of accuracy on KDDTrain+ dataset.

Classifier	Normal	DOS	Probing	R2L	U2R
BPNN^9^	91.50	90.94	80.53	48.13	60.00
FC-ANN^9^	93.87	80.32	89.12	89.57	75.58
GA-ANFIS^9^	96.22	96.70	93.18	94.89	83.33
PSO-ANFIS^9^	96.46	95.90	91.35	94.72	85.62
CSO-ANFIS^9^	97.41	96.25	92.51	94.15	**90.26**
Proposed method	**99.87**	**99.92**	**99.79**	**97.79**	80.77

Significant values are in bold.

**Table 11:** Comparison of CART, ANFIS, and Proposed Method on KDDTest+ Dataset.

Category	Precision (%)	Recall (%)	F1-score(%)	DR(%)	FAR(%)	ACC(%)
CART	**81.06**	76.23	72.52	76.22	23.77	76.23
ANFIS	60.42	65.93	60.80	65.93	34.07	65.93
Proposed method	80.58	**77.46**	**74.46**	**77.46**	**22.54**	**77.46**

Significant values are in bold.

**Table 12:** Comparison of accuracy with other existing approaches on KDDTrain+ dataset.

Category	SVM^3^	DBN^13^	DBN-SVM^3^	ANN (MLP)^14^	Logistic regression^14^	Naïve Bayes^14^	MVO-ANN^14^	Proposed method
ACC (%)	88.33	89.54	92.84	97.04	92.75	95	98.21	**99.87**

According to the comparison, the detection rate of the proposed method is 4.06% better than CSO-ANFIS^9^. The false alarm rate is 2.31% less than CSO-ANFIS^9^. Except for U2R, in the other four main categories, the ACC (accuracy) is superior to CSO-ANFIS.

According to the comparison of single CART, ANFIS, and the proposed method on the KDDTest+ dataset (some intrusions only exist in this test dataset), except for Precision, other metrics of the proposed method are better than single CART and ANFIS, which means the performance is improved.

Meanwhile, for comparing with other fields’ methods combining DT and ANFIS, we compared the proposed method with the work of Tianhua Chen et al.^15,16^ in medical field. Tianhua Chen et al.^15^ proposed an effective approach combining CART and ANFIS to learn a fuzzy rule base. This method used a decision tree learning mechanism to generate a crisp rule base from the data, and converted it into a fuzzy rule base replaced by a Gaussian membership function. Then, the fuzzy rule base will be the input of ANFIS to optimize. The method is able to show the contributions made by individual rules and explain how a conclusion is reached from the given input. And, in 2022, Tianhua Chen et al.^16^ proposed a novel fuzzy inference system adapting the concept of dominant sets to deal with the uncertainty in dementia and generate interpretable rules of diagnosis. The method adapts the concept of dominant sets to formulate the dementia diagnosis as a pairwise clustering problem. A peeling-off strategy is used to extract a dominant set from the edge-weighted graph. Then, the dominant set is converted into a parameterized fuzzy rule, which is further optimized in a supervised adaptive network-based fuzzy inference framework. Different from the work of Tianhua Chen et al.^15,16^, we connect ANFIS to the front of CART and co-train the proposed method. The fuzzy rules generated by modified ANFIS will be the input of CART. And, after the training process of CART, the loss of $(Y,{Y}^{^{\prime}})$ will backpropagate and optimise the ANFIS-part. Then a new CART will be trained according to the new results of ANFIS-part, as shown in Algorithm 1. After several iterations, the overall architecture will be optimized. So, in the proposed method, the weighted fuzzy rules are used to enhance the depth of CART, which will allow deeper pattern extraction. Meanwhile, the structure of proposed method is also interpretable like the works of Tianhua Chen et al.^15,16^, which will help use to interpret the fuzzy characteristics of intrusion behaviours.

To conclude, there are two important experiments conducted in the study. The first one is the interpretable, deeper structure based on ANFIS and CART. In this experiment, a connection and co-training mechanism of ANFIS and CART is proposed. It can generate an interpretable and deeper model than ANFIS and CART, which is useful to read the deeper and fuzzy intrusion patterns. The second one is the efficiency improvement of the proposed algorithm. In this study, we used Pearson correlation analysis to select 22 attributes that are highly related to the target as the experimental attributes. And, for determining the minimum interval division of attributes, standard deviation, and the proposed adaptive K-means algorithm are used. The adaptive K-means is proposed to dynamically minimize the number of each attribute’s intervals.

The performance of the proposed method was also compared with other algorithms: BPNN, FC-ANN, GA-ANFIS, PSO-ANFIS, and CSO-ANFIS. The performance of the proposed method achieved a 99.86% detection rate and 0.14% false alarm rate on the KDDTrain+ dataset, which is better than these methods. And through the comparison of single CART, ANFIS, and the proposed method on the KDDTest+ dataset (some intrusions only exist in this test dataset), the performance of the proposed method is improved.

## Supplementary Information

Supplementary Information.

## Data Availability

The NSL-KDD dataset in this study is available in the Canadian Institute for Cybersecurity repository (https://www.unb.ca/cic/datasets/nsl.html). All data generated during this study are included in this published article (and its Supplementary Information files).
