# Supplementary material for: A hybrid interpretable deep structure based on adaptive neuro-fuzzy inference system, decision tree, and K-means for intrusion detection
Source: Sci Rep. 2022 Dec 1;12:20770. doi: 10.1038/s41598-022-23765-x (PMC9715629; doi:10.1038/s41598-022-23765-x)
Supplement: Supplementary file 1 — Supplementary Information. [file 41598_2022_23765_MOESM1_ESM.pdf]

Supplementary Table S1. The Generated Rules with Four Attributes of Proposed method's ANFIS-part

rule_num	duration	count	dst_host_count	dst_host_srv_count
rule0	0	0	0	0
rule1	0	0	0	1
rule2	0	0	0	2
rule3	0	0	0	3
rule4	0	0	1	0
rule5	0	0	1	1
rule6	0	0	1	2
rule7	0	0	1	3
rule8	0	0	2	0
rule9	0	0	2	1
rule10	0	0	2	2
rule11	0	0	2	3
rule12	0	1	0	0
rule13	0	1	0	1
rule14	0	1	0	2
rule15	0	1	0	3
rule16	0	1	1	0
rule17	0	1	1	1
rule18	0	1	1	2
rule19	0	1	1	3
rule20	0	1	2	0
rule21	0	1	2	1
rule22	0	1	2	2
rule23	0	1	2	3
rule24	0	2	0	0
rule25	0	2	0	1
rule26	0	2	0	2
rule27	0	2	0	3
rule28	0	2	1	0
rule29	0	2	1	1
rule30	0	2	1	2
rule31	0	2	1	3
rule32	0	2	2	0
rule33	0	2	2	1
rule34	0	2	2	2
rule35	0	2	2	3
rule36	0	3	0	0
rule37	0	3	0	1
rule38	0	3	0	2
rule39	0	3	0	3
rule40	0	3	1	0
rule41	0	3	1	1
rule42	0	3	1	2

rule43	0	3	1	3
rule44	0	3	2	0
rule45	0	3	2	1
rule46	0	3	2	2
rule47	0	3	2	3
rule48	1	0	0	0
rule49	1	0	0	1
rule50	1	0	0	2
rule51	1	0	0	3
rule52	1	0	1	0
rule53	1	0	1	1
rule54	1	0	1	2
rule55	1	0	1	3
rule56	1	0	2	0
rule57	1	0	2	1
rule58	1	0	2	2
rule59	1	0	2	3
rule60	1	1	0	0
rule61	1	1	0	1
rule62	1	1	0	2
rule63	1	1	0	3
rule64	1	1	1	0
rule65	1	1	1	1
rule66	1	1	1	2
rule67	1	1	1	3
rule68	1	1	2	0
rule69	1	1	2	1
rule70	1	1	2	2
rule71	1	1	2	3
rule72	1	2	0	0
rule73	1	2	0	1
rule74	1	2	0	2
rule75	1	2	0	3
rule76	1	2	1	0
rule77	1	2	1	1
rule78	1	2	1	2
rule79	1	2	1	3
rule80	1	2	2	0
rule81	1	2	2	1
rule82	1	2	2	2
rule83	1	2	2	3
rule84	1	3	0	0
rule85	1	3	0	1
rule86	1	3	0	2
rule87	1	3	0	3

rule88	1	3	1	0
rule89	1	3	1	1
rule90	1	3	1	2
rule91	1	3	1	3
rule92	1	3	2	0
rule93	1	3	2	1
rule94	1	3	2	2
rule95	1	3	2	3
rule96	2	0	0	0
rule97	2	0	0	1
rule98	2	0	0	2
rule99	2	0	0	3
rule100	2	0	1	0
rule101	2	0	1	1
rule102	2	0	1	2
rule103	2	0	1	3
rule104	2	0	2	0
rule105	2	0	2	1
rule106	2	0	2	2
rule107	2	0	2	3
rule108	2	1	0	0
rule109	2	1	0	1
rule110	2	1	0	2
rule111	2	1	0	3
rule112	2	1	1	0
rule113	2	1	1	1
rule114	2	1	1	2
rule115	2	1	1	3
rule116	2	1	2	0
rule117	2	1	2	1
rule118	2	1	2	2
rule119	2	1	2	3
rule120	2	2	0	0
rule121	2	2	0	1
rule122	2	2	0	2
rule123	2	2	0	3
rule124	2	2	1	0
rule125	2	2	1	1
rule126	2	2	1	2
rule127	2	2	1	3
rule128	2	2	2	0
rule129	2	2	2	1
rule130	2	2	2	2
rule131	2	2	2	3
rule132	2	3	0	0

rule133	2	3	0	1
rule134	2	3	0	2
rule135	2	3	0	3
rule136	2	3	1	0
rule137	2	3	1	1
rule138	2	3	1	2
rule139	2	3	1	3
rule140	2	3	2	0
rule141	2	3	2	1
rule142	2	3	2	2
rule143	2	3	2	3
rule144	3	0	0	0
rule145	3	0	0	1
rule146	3	0	0	2
rule147	3	0	0	3
rule148	3	0	1	0
rule149	3	0	1	1
rule150	3	0	1	2
rule151	3	0	1	3
rule152	3	0	2	0
rule153	3	0	2	1
rule154	3	0	2	2
rule155	3	0	2	3
rule156	3	1	0	0
rule157	3	1	0	1
rule158	3	1	0	2
rule159	3	1	0	3
rule160	3	1	1	0
rule161	3	1	1	1
rule162	3	1	1	2
rule163	3	1	1	3
rule164	3	1	2	0
rule165	3	1	2	1
rule166	3	1	2	2
rule167	3	1	2	3
rule168	3	2	0	0
rule169	3	2	0	1
rule170	3	2	0	2
rule171	3	2	0	3
rule172	3	2	1	0
rule173	3	2	1	1
rule174	3	2	1	2
rule175	3	2	1	3
rule176	3	2	2	0
rule177	3	2	2	1

rule178	3	2	2	2
rule179	3	2	2	3
rule180	3	3	0	0
rule181	3	3	0	1
rule182	3	3	0	2
rule183	3	3	0	3
rule184	3	3	1	0
rule185	3	3	1	1
rule186	3	3	1	2
rule187	3	3	1	3
rule188	3	3	2	0
rule189	3	3	2	1
rule190	3	3	2	2
rule191	3	3	2	3

0-3 represent the interval of attribute

Supplementary Table S2. The Coefficient with Four Attributes of Each Rule of Proposed Method's ANFIS-part

rule number	duration	count	dst_host_count	dst_host_srv_count	r
rule0	-0.001081373	0.001768952	-0.054062329	0.001218294	0.02635674
rule1	0.139038563	0.077832215	-0.200930342	0.799063981	-0.385030448
rule2	0.132066861	-0.285668701	-1.924451113	0.634215117	-0.586823046
rule3	0.160386488	-0.529788375	-2.695405245	-0.505216897	-0.904096603
rule4	-0.033822022	0.799601138	0.955424905	-0.043199532	0.11039789
rule5	-1.860505342	4.401194572	-2.359276533	-0.784082592	-0.67827636
rule6	-0.198397279	2.083471298	2.683088303	-1.102190018	-0.312144995
rule7	0.214376464	-0.311915219	-2.610447645	-1.874673605	-1.05183804
rule8	-0.216854125	-0.372936517	-12.3175993	-0.938369393	-1.217832565
rule9	-0.590529144	1.327121377	-7.090093613	-0.196419567	-0.955979288
rule10	-0.098307095	1.08185935	2.751200676	0.373473167	-0.148595378
rule11	-0.00114681	0.528308868	0.015081241	-0.788546562	-0.488345027
rule12	-0.001322923	0.047712218	0.03107987	-0.006127751	0.019351611
rule13	0.191806406	0.246356755	-0.448630273	0.012798227	-0.51706028
rule14	0.181211203	-0.132366851	-2.217318773	-0.781094968	-0.753827512
rule15	0.212443754	-0.390309364	-3.019026041	-1.079549193	-0.943899572
rule16	-0.027433943	-2.640571356	-7.8497715	-0.484213769	-0.769171417
rule17	-1.548238754	1.164277434	-11.49091148	-1.900151134	-1.609097362
rule18	-0.12165805	2.259680986	2.23331809	-1.730063081	-0.379487753
rule19	0.270934731	-0.12045256	-2.936773539	-2.546766281	-1.094863534
rule20	-0.154171333	-13.35506916	-22.51743507	-1.991807699	-2.24693799
rule21	-0.460084617	-18.02322006	-32.57452774	0.634644508	-3.538602352
rule22	-0.067587785	1.483944416	3.617157936	0.663432539	-0.068473667
rule23	0.025569208	0.921133816	1.24286294	0.0695903	-0.368428022
rule24	0.001383552	0.001983907	0.000504626	-0.002595596	0.001990248
rule25	0.017937519	0.425003797	1.896654129	1.247033834	-0.342300326
rule26	-0.008393357	0.082253821	0.375665635	0.423784912	-0.567577243
rule27	0.004580019	0.229592174	0.184964687	0.550007284	-0.598857939
rule28	-0.016498374	-2.355172396	-6.56633234	-0.358063519	-0.652447283
rule29	-0.142536968	2.193591833	4.833705425	1.25861001	-0.142307788
rule30	-0.011521723	1.220077634	3.852017641	0.185011312	-0.369526386
rule31	0.05202603	0.425407559	1.301709056	0.359811991	-0.657411456
rule32	-0.120529056	42.22142029	74.76231384	2.662210464	7.477144241
rule33	-0.236752048	-19.1728344	-54.53039551	-3.255004644	-5.887291431
rule34	-0.074017659	0.818728805	2.621268749	0.377600431	-0.252075732
rule35	-0.061304629	0.606279135	0.895581007	-0.582393706	-0.429499507
rule36	-1.43E-12	5.29E-10	3.20E-10	3.54E-10	5.63E-11
rule37	0.22649318	0.145881921	1.358865499	-0.239084437	-0.590341568
rule38	0.19332014	-0.258645356	-0.410698742	-1.16710794	-0.833642244
rule39	0.214045718	0.059079777	-0.383914173	-1.07366538	-0.822971761
rule40	3.32E-09	0.010082212	0.007777808	0.002924394	0.001030695
rule41	0.284090012	0.27335152	3.002543688	-0.196016133	-0.569669902
rule42	0.247772858	-0.166586131	1.006505728	-1.313096523	-0.849506676
rule43	0.268794537	0.179216802	1.054638505	-1.182042122	-0.835316658
rule44	3.35E-09	0.002126397	0.00232107	0.000195794	0.000232108
rule45	0.023408396	0.788968742	3.75501132	1.071406841	-0.172280014
rule46	0.007694576	0.463404953	2.705916882	0.450632095	-0.317585111
rule47	0.011284348	0.837607026	2.844821692	0.688829958	-0.29522258
rule48	-0.004519064	0.001422714	-0.009442911	-0.003010672	0.004939981
rule49	0.250881672	0.043879054	-0.378223062	1.1298666	-0.506572962
rule50	0.245057687	-0.479167342	-2.898801565	-0.233154982	-0.939166307
rule51	0.28939873	-0.881568313	-4.05624485	-0.596299052	-1.216325521
rule52	-0.039527472	1.102188468	1.394570112	-0.045735218	0.160385922
rule53	-2.523619413	5.876322269	-4.082207203	-1.113071918	-0.927957833
rule54	-0.233344123	2.893269539	3.358667374	-1.705601811	-0.404224843
rule55	0.365388602	-0.571603179	-3.91358304	-2.762370825	-1.435795069
rule56	-0.096449003	-0.389258802	-11.86398697	-1.048708439	-1.168071508
rule57	-0.648975015	1.84504509	-9.428413391	-0.262226313	-1.240134835
rule58	-0.122723676	1.526170611	3.675691128	0.473174334	-0.173608884
rule59	0.019227743	0.716616571	0.433105946	-0.700147212	-0.594456077
rule60	-0.004726472	0.01691396	-0.002994359	-0.004693782	0.008491442
rule61	0.327764302	0.25034219	-0.855771124	0.087053426	-0.633770883
rule62	0.316109389	-0.302399963	-3.430533409	-1.188510656	-1.026818037
rule63	0.364781171	-0.722803831	-4.575351238	-1.583468676	-1.298059225
rule64	-0.032212023	-3.443399429	-10.25354004	-0.63297838	-1.003252029
rule65	-2.088859797	1.696448684	-16.20996094	-2.723174572	-2.189775467

rule66	-0.122992493	3.100889921	2.821798563	-2.524126053	-0.493132323
rule67	0.447178274	-0.339542449	-4.412407398	-3.696424007	-1.502890944
rule68	-0.119420961	-30.40664101	-70.93028259	-3.970664024	-7.081583023
rule69	-0.494559944	-31.49012566	-58.61354065	0.432494402	-6.206771851
rule70	-0.078758582	1.9283216	4.559586525	0.620995343	-0.095715322
rule71	0.057522438	1.396550536	2.190544605	0.559102178	-0.424201638
rule72	0.001868544	0.002623487	0.000763689	-0.003362291	0.00275181
rule73	0.071997277	0.561711729	2.681076288	1.727264643	-0.422927201
rule74	0.033724435	0.07018654	0.485408992	0.538267136	-0.746230483
rule75	0.055048671	0.245709956	0.230158791	0.700734556	-0.790516555
rule76	-0.020229546	-3.153582335	-8.750577927	-0.476223409	-0.869057298
rule77	-0.148974016	3.224707127	7.351079941	1.864966154	-0.07310807
rule78	0.02647179	1.698243618	5.471745491	0.290784985	-0.44276306
rule79	0.12106391	0.516711295	1.843843341	0.517369628	-0.854248047
rule80	-0.151729286	-27.01334381	-69.70384979	-3.539674282	-6.969000816
rule81	-0.29708153	-35.90541458	-91.80428314	-5.370013237	-9.739413261
rule82	-0.091568582	1.162162662	3.780166626	0.605331957	-0.292876184
rule83	-0.071819089	0.854179382	1.447196364	-0.656881988	-0.534337461
rule84	-1.95E-12	7.01E-10	4.25E-10	4.70E-10	7.48E-11
rule85	0.387769043	0.166923299	1.790740252	-0.59646982	-0.796040595
rule86	0.339234829	-0.41465345	-0.768332541	-1.938379526	-1.146881223
rule87	0.372258693	0.00825736	-0.711018741	-1.829311728	-1.130831003
rule88	4.57E-09	0.013418086	0.010364574	0.003880391	0.001371589
rule89	0.468460143	0.352972358	4.189837933	-0.413980484	-0.740005851
rule90	0.415261477	-0.278165549	1.304824233	-2.026432991	-1.143274188
rule91	0.448907703	0.178744435	1.396084189	-1.867810249	-1.121955037
rule92	4.47E-09	0.03839048	0.038322512	0.00361887	0.003832253
rule93	0.055013657	1.54572022	5.772963524	1.755168796	-0.141005889
rule94	0.032006182	0.857899666	4.040636063	0.764200628	-0.371578753
rule95	0.038780957	1.343895316	4.23221302	1.10833478	-0.341175795
rule96	-0.004151078	-2.14E-05	-9.54E-05	-8.71E-05	-0.001094347
rule97	-0.002720724	-0.448085606	-1.988174677	0.298294693	-0.842814147
rule98	-0.041786838	-0.963900745	-4.398100853	-1.034447908	-1.171277881
rule99	-0.00346219	-1.126066446	-4.983433247	-0.963133991	-1.287170887
rule100	0.002068437	1.48E-05	0.007390857	2.78E-05	0.000722609
rule101	-0.691599369	1.277299643	3.771636248	1.371480465	-0.46404174
rule102	-0.323258072	0.753013194	2.017140865	-0.15969345	-0.745896995
rule103	0.024319593	-0.802916229	-2.066513777	-0.104076743	-1.203748345
rule104	0.12830852	0.000990826	0.261510909	0.002312399	0.026151128
rule105	-0.298324764	1.093824863	4.204276085	1.41082406	-0.16329959
rule106	-0.280432194	0.828949153	3.078096151	0.704080045	-0.328266531
rule107	-0.19813332	0.553813577	1.837641597	0.824058235	-0.464335352
rule108	-0.003877367	-2.00E-05	-8.83E-05	-8.14E-05	-0.001022717
rule109	0.064386941	-0.278280854	-2.443759918	-0.480077893	-0.902121484
rule110	0.023635656	-0.805821121	-4.918300152	-1.847494125	-1.238936067
rule111	0.066248633	-0.977693081	-5.484055996	-1.768696189	-1.351808429
rule112	0.001592415	9.61E-06	0.005804855	2.08E-05	0.000565126
rule113	-0.545165539	1.557469606	3.666261911	0.555639088	-0.488375396
rule114	-0.236640856	1.022432208	1.739607215	-1.01372695	-0.78938514
rule115	0.099953808	-0.605848789	-2.536178827	-0.962924957	-1.265654445
rule116	0.021737773	0.000154318	0.043381479	0.00032846	0.004338145
rule117	-0.257518888	1.346171737	4.284375668	1.04075253	-0.153720364
rule118	-0.242931396	1.077871203	3.128784418	0.318505555	-0.322331011
rule119	-0.16267693	0.785520494	1.842778683	0.439055264	-0.462623179
rule120	3.59E-07	7.44E-09	5.09E-08	4.02E-07	3.25E-07
rule121	-0.199718088	0.205600634	1.4254179	1.142837644	-0.57694602
rule122	-0.238094524	-0.254577249	-0.620090723	0.047523368	-0.858030379
rule123	-0.220653951	-0.025890736	-0.712036729	0.22312066	-0.870306194
rule124	0.000409024	1.54E-06	0.001442077	5.40E-06	0.000144257
rule125	-0.257567465	0.790305436	4.124883175	0.921872616	-0.51586771
rule126	-0.262752473	0.285182744	1.919414639	-0.357197881	-0.828461707
rule127	-0.202736869	0.080827564	0.538424194	-0.199167341	-0.967324555
rule128	4.88E-05	1.14E-05	0.000195872	7.54E-07	1.96E-05
rule129	-0.281838	0.939811409	3.913063765	1.113680959	-0.238107562
rule130	-0.294478238	0.689358354	2.847950459	0.507178366	-0.391237527
rule131	-0.284246773	0.817768276	2.439880371	0.659427166	-0.42676568
rule132	-2.34E-12	2.60E-11	-3.22E-11	1.55E-11	-2.90E-12
rule133	0.080208391	-0.112983108	0.640508294	-1.061910629	-0.891350865

rule134	0.032423679	-0.659290731	-1.762480736	-2.323480129	-1.220976353
rule135	0.059984226	-0.261609197	-1.721788287	-2.219854832	-1.207036138
rule136	-2.55E-12	5.44E-09	4.14E-09	1.58E-09	5.49E-10
rule137	0.109747104	0.0300116	2.788052559	-0.945368052	-0.854665279
rule138	0.057223402	-0.590050817	0.054780204	-2.42775631	-1.235571265
rule139	0.084819868	-0.132582933	0.142871946	-2.289439201	-1.214559078
rule140	-1.66E-12	1.52E-08	1.53E-08	1.44E-09	1.52E-09
rule141	-0.174044609	0.738465846	4.232902527	0.987543821	-0.276816636
rule142	-0.197635144	0.43904826	2.956787348	0.28764087	-0.458290726
rule143	-0.19387579	0.775994062	3.00496316	0.439241767	-0.442843378
rule144	-0.002012988	-5.18E-06	-0.000498161	-0.000134919	-0.000264649
rule145	0.332277179	1.528301477	10.3962822	5.348337173	0.449001253
rule146	0.291928738	0.977288127	7.894379139	3.957846642	0.104648538
rule147	0.32136488	1.448859096	8.438435555	4.553383827	0.193117037
rule148	-0.062932283	-0.00028249	-0.07204517	-0.000585873	-0.007238658
rule149	-0.787251651	1.238612413	8.908841133	4.177410126	0.060095165
rule150	0.078895994	0.621285737	7.10497427	2.595172167	-0.231935546
rule151	0.386761248	1.155265689	7.933054447	3.18535471	-0.114654452
rule152	0.072855644	0.000243537	0.062223762	0.000488029	0.006222373
rule153	-0.519348264	1.116298914	5.376068592	2.247624874	-0.074370012
rule154	-0.042074516	0.821151733	4.656406879	1.519568682	-0.201288447
rule155	0.032181624	1.179224133	4.906630516	1.818805099	-0.158381179
rule156	-0.001712054	-4.40E-06	-0.000423688	-0.000114749	-0.000225085
rule157	0.406323433	1.86816299	10.72436523	4.865225315	0.479352862
rule158	0.36438635	1.305169344	8.157360077	3.43857193	0.126774207
rule159	0.396506995	1.786006451	8.742208481	4.058995247	0.220422089
rule160	-0.051362179	-0.0002305	-0.058781255	-0.000478731	-0.005907141
rule161	-0.558796346	1.599603772	9.312674522	3.58443594	0.096339598
rule162	0.172738671	0.968842208	7.293314457	1.962142467	-0.219135985
rule163	0.467675239	1.512906432	8.144284248	2.575136423	-0.098575927
rule164	-0.234258235	-0.001076021	-0.274922997	-0.00215626	-0.027492339
rule165	-0.396524787	1.404635549	5.699707985	1.937833428	-0.03712216
rule166	-0.001937766	1.105009675	4.858265877	1.19314456	-0.176892996
rule167	0.069842286	1.470051885	5.124083996	1.501168013	-0.132052377
rule168	-1.86E-12	2.67E-11	-3.31E-11	1.53E-11	-3.01E-12
rule169	0.062985294	0.750344336	4.695841789	2.388947248	-0.291291595
rule170	0.026967481	0.270852387	2.591834545	1.260462642	-0.58098191
rule171	0.04048967	0.715738356	2.805474997	1.588949561	-0.541736484
rule172	-0.02435345	-0.000109517	-0.027981641	-0.000219464	-0.002798159
rule173	-0.019379646	0.621891141	4.910216808	1.360837579	-0.510390222
rule174	0.050177414	0.076765157	2.640815258	0.045022875	-0.832581043
rule175	0.102403983	0.58518213	2.890043497	0.383351952	-0.788845837
rule176	-0.146197721	-0.000657474	-0.167984858	-0.001317527	-0.016798459
rule177	-0.25597772	0.91456902	3.982939959	1.213005424	-0.282657206
rule178	-0.078414552	0.648569882	3.077502251	0.58798033	-0.421701282
rule179	-0.068998419	0.998229861	3.146682501	0.80227977	-0.402373135
rule180	-1.91E-12	2.77E-11	-3.46E-11	1.65E-11	-3.12E-12
rule181	0.293499172	-0.821864784	-3.05713439	-3.008848667	-1.440822244
rule182	0.248123869	-1.377656698	-5.477371693	-4.271422863	-1.77315712
rule183	0.27527082	-0.953180373	-5.584542274	-4.221579075	-1.778220177
rule184	-2.04E-12	3.19E-11	-3.92E-11	1.82E-11	-3.62E-12
rule185	0.373742163	-0.745830357	-1.031740308	-3.08911109	-1.443919063
rule186	0.323927075	-1.379240513	-3.796514273	-4.580729961	-1.829644799
rule187	0.351587474	-0.877773046	-3.846138239	-4.490730762	-1.826408386
rule188	-1.29E-12	2.26E-11	-2.58E-11	1.29E-11	-2.35E-12
rule189	0.03230501	0.496430099	2.980915308	0.247499153	-0.49947843
rule190	0.010782043	0.188266158	1.678574681	-0.464001805	-0.684982896
rule191	0.015415906	0.548541963	1.671357512	-0.324017853	-0.676208138

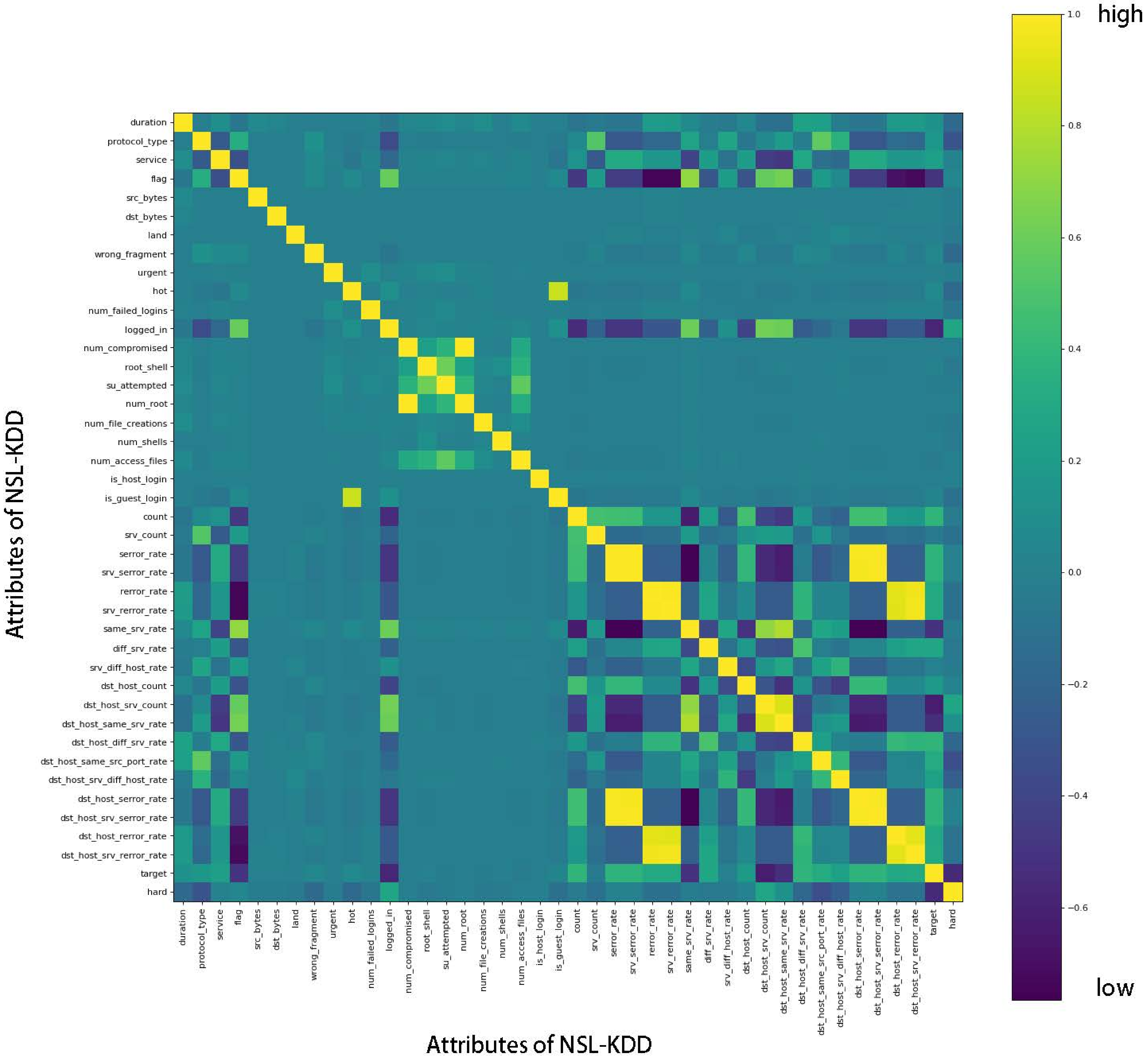

Supplementary Figure S1. Correlation Matrix between Attributes of NSL-KDD Dataset. This figure is generated by python 3.7. https://www.kaggle.com/code/lsegily/attribute-selection/notebook.

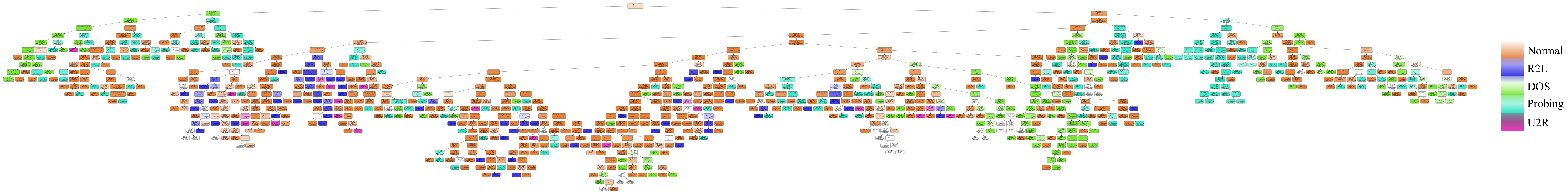

Supplementary Figure S2. The Generated CART-part of proposed method. This figure is generated by python 3.7 based on the 80% NSL-KDD Train+ dataset. The rules in the figure is the generated rules by the ANFIS-part of proposed method. The first attribute of each node represents the division condition. The gini attribute of each node is the gini index of each node. The sample attribute of each node is the number of data in this node. The value attribute of each node repersents the amount of data in five main categories. The class attribute of each node represents which of the five main categories the node should belong to.
